# Mitochondrial protection impairs BET bromodomain inhibitor-mediated cell death and provides rationale for combination therapeutic strategies

**DOI:** 10.1038/cddis.2015.352

**Published:** 2015-12-10

**Authors:** E Lasorsa, M Smonksey, J S Kirk, S Rosario, F J Hernandez-Ilizaliturri, L Ellis

**Affiliations:** 1 Department of Pharmacology and Therapeutics, Roswell Park Cancer Institute, Buffalo, NY, USA; 2 Department of Molecular Pharmacology and Cancer Therapeutics, State University of New York at Buffalo, Buffalo, NY, USA; 3 Department of Medicine, Roswell Park Cancer Institute, Buffalo, NY, USA

## Abstract

Inhibitors of the bromodomain and extraterminal domain family (BETI) have recently entered phase I clinical trials. In patients with advanced leukemia's, potent antileukemia activity was displayed with minimum dose-limiting toxicity. In preclinical models of hematological malignancies, including aggressive B-cell lymphomas, BETI induced cell-cycle arrest and apoptosis. However, the underlying cell death mechanisms are still not well understood. Dissecting the mechanisms required by BETI to mediate cell death would provide strong direction on how to best utilize BETI to treat patients with aggressive hematological malignancies. Herein, we provide understanding of the molecular mechanisms underlying BETI-mediated cell death using I-BET762. Induction of cell death occurred in primary murine and human B-cell lymphomas through apoptosis. Genetic dissection using E*μ*-*myc* B-cell lymphoma compound mutants demonstrated that I-BET762-induced apoptosis does not require the p53 pathway. Furthermore, deletion of Apaf1, and thus the absence of a functional apoptosome, is associated with a delayed drug response but do not provide long-term resistance. Prolonged treatment of this model in fact fails to suppress the therapeutic efficacy of the drug and is associated with biochemical features of autophagy. However, lack of mitochondrial permeability completely inhibited I-BET762-mediated tumor cell death, indicating mitochondrial damage as key events for its activity. Combination of I-BET762 with BH3-only mimetics ABT-263 or obatoclax, restored sensitivity to I-BET762 lymphoma killing; however, success was determined by expression of Bcl-2 family antiapoptotic proteins. Our study provides critical insight for clinical decisions regarding the appropriate strategy for using BETI as a single agent or in combination to treat patients with aggressive B-cell lymphomas.

Aggressive hematological malignancies including B-cell lymphomas commonly involve deregulation of the *Myc* oncogenic activity. Increased *Myc* oncogenic action via gene rearrangement is a hallmark of Burkitt lymphoma and found in ~10% of diffuse large B-cell lymphoma (DLBCL). More frequent in DLBCL is the upregulation of Myc protein expression, which has been identified in 25–30% of patients.^1, 2^ Increased Myc expression is correlated with poorer outcome in patients treated with standard of care therapies including rituximab and chemotherapy. To add complexity to the clinical management for these aggressive DLBCL is the simultaneous expression of antiapoptotic proteins including Bcl-2, Bcl-X or Mcl-1.^1, 2^ Owing to inferior responses of these patients to standard care of treatment, novel therapeutic approaches are urgently required.

Recently, inhibitors of bromodomain and extraterminal domain (BET) proteins have shown potent antagonism of Myc transcriptional activity and protein expression, primarily through manipulation of the BET bromodomain protein BRD4. Two classes of BET inhibitors (BETI), the benzodiazapenes and quinolones, have been recently shown to exhibit significant *in vitro* and *in vivo* antitumor activity in multiple tumor types including lung cancer, prostate cancer, neuroblastoma and various hematological malignancies including B-cell lymphoma.^3, 4, 5, 6, 7, 8, 9, 10, 11^ Excitingly, recent data from a phase I trial of the BET inhibitor OTX-015 displayed potent single-agent antileukemic activity with minimum toxicity.^12^

Antitumor mechanisms induced by BET inhibitors are currently not well understood. Most critical is gaining a key understanding of pathways required by BET inhibitors to mediate apoptosis or cell death. The focus of this study was to identify key proteins and pathways required for the clinical compound I-BET762^13^ to induce tumor cell killing. For this, we took advantage of a range of independently derived murine E*μ*-*myc* B-cell lymphomas, and human isogenic B-cell lymphoma cell lines either sensitive or resistant to rituximab and chemotherapy. Our data indicate that I-BET762-induced cell death is independent of p53 and apoptosome pathways. Conversely, protection of mitochondrial integrity diminished I-BET762 antitumoral activity, thus demonstrating the importance of mitochondrial damage as a key event in I-BET762-mediated apoptosis. Interestingly, chemical suppression of antiapoptotic proteins restored lymphoma killing by I-BET762. Our study provides critical insight for clinical decisions regarding precision medicine strategies for using BET inhibitors as a single agent or in combination to treat patients with aggressive B-cell lymphomas.

## Results

### I-BET762 induces apoptosis in mouse and human models of B-cell lymphoma

**Figure 1:**
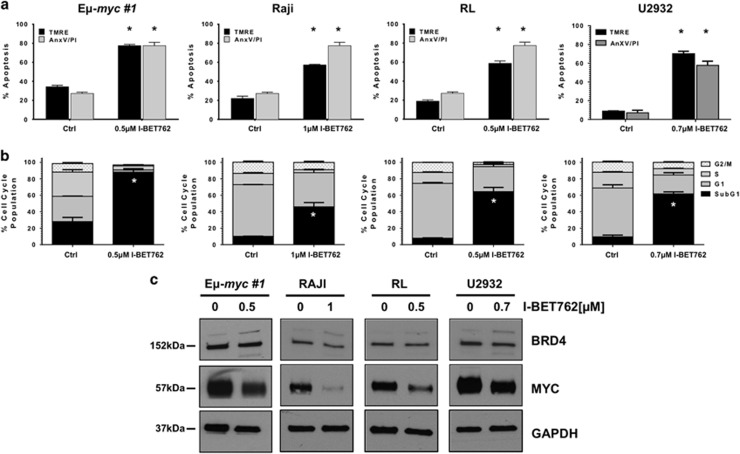
I-BET762 induces apoptosis in mouse and human models of B-cell lymphoma. (**a** and **b**) E*μ*-*myc*, RAJI, RL and U2932 B-cell lymphomas were treated with respective LD_70_ concentrations of I-BET762 for 48 or 240 h respectively. Apoptosis was assessed by flow cytometry analysis of TMRE, surface exposure of phosphotidylserine (annexin V staining) and cell-cycle analysis. Each point represents the mean value ±S.E. of three individual experiments, *P<0.05. (**c**) Western blot analysis on whole-cell lysates prepared from mouse and human lymphoma cell lines treated, respectively, for 24 and 72 h with corresponding LD_70_ concentrations of I-BET762 or DMSO. The protein expression of BRD4 and Myc was assessed. GAPDH was used as a loading control

To assess the sensitivity of different subtypes of B-cell lymphoma to BET inhibition, murine E*μ*-*myc* and human B-cell lymphomas were exposed to increasing concentrations of I-BET762 over time as indicated (Supplementary Figure S1). As detected by propidium iodide (PI) uptake, exposure to I-BET762 resulted in loss of plasma membrane integrity with a dose- and time-dependent effect (Supplementary Figure S1). The calculated concentration of I-BET762 resulting in 70% cell death (LD_70_) at 48 h of E*μ*-*myc* lymphomas was 0.5 *μ*M. In all the human B-cell lymphoma cell lines tested, the kinetic of response resulted delayed, and a significant cell death detectable only after 6–10 days of treatment (Figure 1a). Nevertheless, the sensitivity of all the three models is very high with LD_70_s varying from 500 to 1000 nM at day 10 (Supplementary Figure S1). LD_70_ concentrations of I-BET762 were sufficient to induce hallmark features of apoptosis, including loss of mitochondrial membrane potential, caspase activation, loss of clonogenic potential (E*μ*-*myc* lymphomas), increased cell surface exposure of phosphotidylserine and DNA fragmentation (Figures 1a and b, Supplementary Figure S1 and Supplementary Tables S1 and S2). I-BET762 exposure did not result in loss of BRD4 protein expression, but did induce marked reductions in Myc protein expression in each cell line (Figure 1c).

### I-BET762-mediated apoptosis is independent of a functional p53 pathway

**Figure 2:**
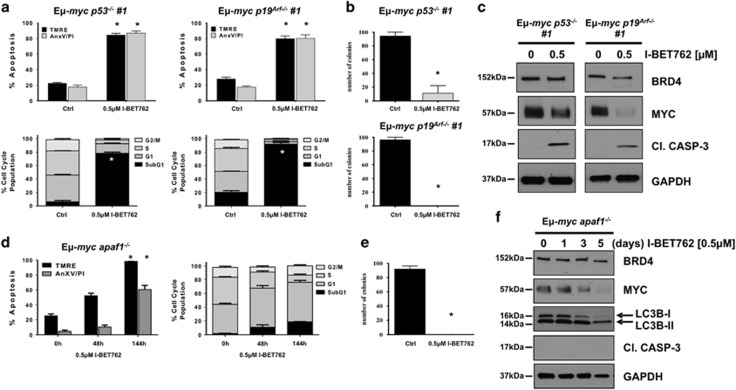
I-BET762-mediated apoptosis is independent from p53 and apoptosome signaling. E*μ*-*myc/p53*^−/−^ and E*μ*-*myc/p19*^*Arf*−/−^ lymphomas were treated for 48 h with 0.5 *μ*M I-BET762. (a) Apoptosis was assessed by flow cytometric analysis of TMRE, surface exposure of phosphotidylserine (annexin V staining) and cell-cycle analysis. (**b**) Clonogenic assay on E*μ*-*myc/p53*^−/−^ and E*μ*-*myc/p19*^*Arf*−/−^ lymphomas after being pretreated for 48 h with DMSO or I-BET762 and then seeded in soft agar. Colonies have been counted after 12 days in culture. Each point represents the mean ±S.E. value of three individual experiments, *P<0.05. (**c**) Western blot analysis on whole-cell lysates prepared from E*μ*-*myc/p53*^−/−^ and E*μ*-*myc/p19*^*Arf*−^ lymphomas treated for 24 h with 0.5 *μ*M of I-BET762 or DMSO. Expression of BRD4, Myc and activated caspase-3 was assessed. GAPDH was used as a loading control. (**d**) E*μ*-*myc/Apaf1*^−/−^ lymphomas were treated with 0.5*μ*M for indicated times. Apoptosis was assessed by flow cytometric analysis of TMRE, surface exposure of phosphotidylserine (annexin V staining) and cell-cycle analysis. (**e**) Clonogenic assay on E*μ*-*myc/Apaf1*^−/−^ lymphomas after being pretreated for 48 h with DMSO or I-BET762 and then seeded in soft agar. Colonies have been counted after 12 days in culture. Each point represents the mean value ±S.E. of three individual experiments, *P<0.05. (**f**) Western blot analysis on whole-cell lysates prepared from E*μ*-*myc/Apaf1*^−/−^ lymphomas treated for 24 h with 0.5 *μ*M of I-BET762 or DMSO. Expression of BRD4, Myc, LC3 and activated caspase-3 was assessed. GAPDH was used as a loading control

Numerous chemotherapy agents rely on an intact p53 pathway for optimal activity,^14^ and p53 dependency for BET bromodomain inhibitor-mediated apoptosis may be cell context dependent.^15, 16^ We therefore examined I-BET762 sensitivity of E*μ*-*myc/p53*^−/−^ and E*μ*-*myc/p19*^*arf*−/−^ lymphomas. While the E*μ*-*myc/p53*^−/−^ model lacks the tumor suppressor gene *p53*, the E*μ*-*myc/p19*^*Arf*−/−^ model retains a wild type but less active p53 protein. The deletion of the *p19*^*Arf*^ gene, orthologous of *p14^Arf^* in humans, in fact leads to an increased degradation of p53 protein by Mdm2 ubiquitin ligase.^17^ LD_70_ treatment of I-BET762 for 48 h induced apoptosis in both genetic compound mutant E*μ*-*myc* lymphomas devoid of p53 signaling, with comparable biochemical features of apoptosis as control E*μ*-*myc* lymphomas, including loss of mitochondrial membrane potential, increased cell surface exposure of phosphotidylserine, loss of clonogenic potential, DNA fragmentation and caspase activation (Figures 2a and b,Supplementary Figure S2 and Supplementary Table S1). Also, in line with control E*μ*-*myc* lymphomas, modest effects of BRD4 protein expression were observed, while Myc protein stability was significantly attenuated following I-BET762 treatment (Figure 2c).

### Lack of functional apoptosome formation delays I-BET762-mediated cell death, and induces molecular changes associated with autophagy

To evaluate the requirements of apoptosome dependence for I-BET762-mediated apoptosis, we took advantage of the E*μ*-*myc*/*Apaf1*^−/−^ model. Deletion of Apaf1 abrogated I-BET762 ability to induce apoptosis at 48 h. A time course experiment revealed that, after 96 and 144 h post I-BET762 treatment, a comparable induction of mitochondrial membrane potential loss could be detected, accompanied by a significant increase in cell death (Figure 2d). However, none of the well-established hallmarks of apoptosis, such as DNA fragmentation or caspase activation, could be detected (Figure 2d and Supplementary Table S3). This significant delay in I-BET762 antitumor activity still demonstrated similar protein expression profiles for BRD4 and Myc in comparison with control E*μ-myc* lymphomas. With this, we determined the ultimate fate of E*μ*-*myc/apaf1*^−/−^ lymphomas following I-BET762 treatment by clonogenic assay. As shown in Figure 2e, clonogenic survival of E*μ*-*myc/apaf1*^−/−^ lymphomas was suppressed, demonstrating I-BET762 ability to induce cell death also in the absence of a functional apoptosome. Furthermore, it has been previously demonstrated that epigenetic-targeted therapies, including inhibitors of histone deacetylases (HDACI), retain the ability to mediate cell death in the absence of a functional apoptosome via induction of autophagy.^18, 19^ One such hallmark of drug-induced autophagy is a conversion of LC3B-I to LC3B-II, which can be detected by western blotting.^20, 21^ We therefore performed a time course treatment of E*μ*-*myc/apaf1*^−/−^ lymphomas with I-BET762 *in vitro* and examined LC3B protein level changes. As shown in Figure 2f, exposure of E*μ*-*myc/apaf1*^−/−^ lymphomas to I-BET762 caused an increase in the ratio of the active form of LC3 (LC3 II) over the inactive (LC3 I). Taken together, these data suggest the involvement of autophagy as alternative mechanism in I-BET762-mediated cell death. However, further studies will be needed to better elucidate a possible role of autophagy in this process.

### Mitochondrial damage is necessary for I-BET762-mediated apoptosis

**Figure 3:**
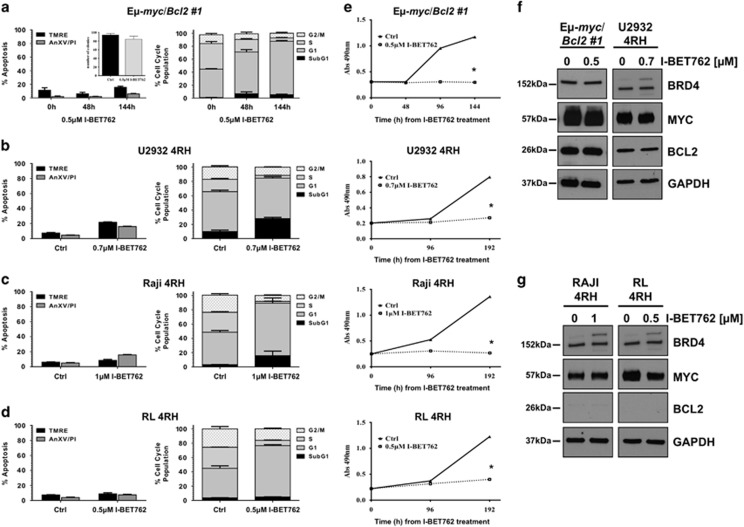
Mitochondrial damage is necessary to induce I-BET762-mediated apoptosis. (**a**) E*μ*-*myc/Bcl-2* lymphomas were treated as indicated with 0.5 *μ*M I-BET762. (Inset) Clonogenic assay with E*μ*-*myc/Bcl-2* lymphomas after being pretreated for 48 h with DMSO or I-BET762 and then seeded in soft agar. Colonies have been counted after 12 days in culture. (**b–d**) Retuximab/chemoresistant human lymphoma models (4RH) were treated for 240 h with indicated concentrations of I-BET762. Apoptosis was assessed by flow cytometric analysis of TMRE, surface exposure of phosphotidylserine (annexin V staining) and cell-cycle analysis. (**e**) MTS assay to evaluate the proliferation rate of all four lymphoma models in the presence or absence of the I-BET762 over time. Each point represents the mean value of three individual experiments ±S.E. (**f**) Western blot analysis on whole-cell lysates prepared from E*μ*-*myc/Bcl-2* cell line treated for 24 h and (**g**) Raji-4RH and RL-4RH lymphomas treated for 72 h with the indicated doses of I-BET762 or DMSO. The expression of indicated proteins was detected with specific antibodies and loading normalization was confirmed by GAPDH probing

Alterations of the antiapoptotic machinery are common genetic alterations observed in B-cell lymphoma patients. The overexpression of prosurvival proteins, such as Bcl-2 or Bcl-6, through translocation or amplification, leads to mitochondrial damage protection and often correlates with drug resistance and poorer prognosis in patients with B-cell lymphoma.^1^ To determine the importance of mitochondrial damage in BETI-mediated apoptosis, we took advantage of E*μ*-*myc/bcl-2* lymphomas and human B-cell lymphoma cell lines refractory to standard care of therapy (U2932-4RH, Raji-4RH and RL-4RH), and investigated their sensitivity to I-BET762 *in vitro*. As shown in Figure 3a and Supplementary Figure S3a, E*μ*-*myc/bcl-2* lymphomas were completely resistant to I-BET762-mediated apoptosis after 48 h exposure. To ensure that overexpression of Bcl-2 did not merely delay apoptosis mediated by I-BET762, E*μ*-*myc/bcl-2* lymphomas were exposed to I-BET762 over 144 h. Lymphomas overexpressing Bcl-2 remained resistant to apoptosis following extended exposure to I-BET762. Although the ectopic overexpression of Bcl-2 suppressed I-BET762-induced apoptosis, inhibition of cell-cycle progression still occurred. This was evident by a decrease of cells in the S phase and concomitant increase of cells in the G_0_/G_1_ phases, as well as cell growth inhibition measured by MTS assay (Figures 3a and e, Supplementary Figure S3a and Supplementary Table S3).

These results were further validated in the Retuximab/chemoresistant human B-cell lymphomas. In particular, in Raji-4RH and RL-4RH models, the multidrug resistance phenotype is associated with the acquisition of dysfunctional mitochondria due to increased levels of MCL-1/Bcl-XL and lower levels of Bak/Bax. However no upregulation of functional multidrug resistance protein pumps could be detected, excluding an increased drug efflux as mechanism of resistance.^22^ Upon 10 days of treatment with I-BET762, all human cell lines displayed no induction of apoptosis as well as no loss of mitochondrial membrane integrity, confirming the importance of mitochondrial damage to achieve drug efficacy (Figures 3b and d). Again, I-BET762 treatment leads to cell-cycle arrest in the G0/G1 phases as well as cell growth inhibition (Figure 3e and Supplementary Table S4). Western blot analysis from all B-cell lymphoma cell lines showed that BRD4, Myc and Bcl-2 protein expression was not altered following I-BET762 treatment over time (Figure 3f).

These data demonstrate that alterations impairing the correct mitochondrial function block the apoptotic process triggered by the I-BET762, altering the response from programmed cell death to growth arrest.

### Overcoming mitochondria protection restores I-BET762 mediated apoptosis

**Figure 4:**
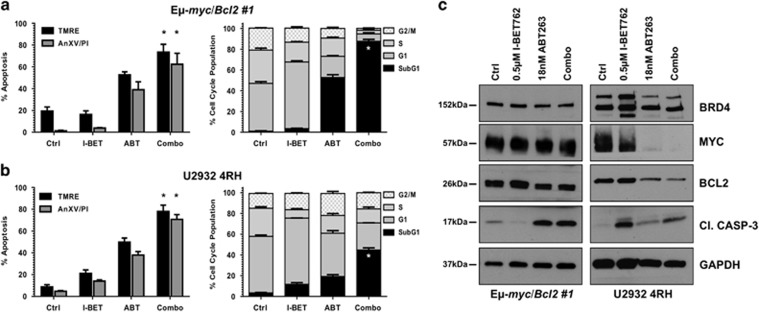
Combination of I-BET762 with ABT-263 increases apoptosis in rituximab/chemoresistant B-cell lymphomas overexpressing Bcl-2. (**a** and **b**) E*μ*-*myc/Bcl-2* and human U2932-4RH lymphomas were treated with indicated concentrations of ABT-263 or I-BET762 for 48 and 240 h, respectively. Apoptosis was assessed by flow cytometry analysis of TMRE, surface exposure of phosphotidylserine (annexin V staining) and cell-cycle analysis. Each point represents the mean ±S.E. value of three individual experiments, *P<0.05. (**c**) Western blot analysis on whole-cell lysates prepared from the E*μ*-*myc/Bcl-2* and human U2932-4RH lymphomas treated for 24 and 72 h, respectively, with the single agents or in combination. The expression of indicated proteins was detected with specific antibodies and loading normalization was confirmed by GAPDH probing

Thus far, our data have indicated the requirement for mitochondrial damage for I-BET762 to mediate apoptosis. In order to find alternative strategies that are able to overcome resistance due to mitochondrial block, we tested the ability of BH3 mimetic compounds to restore mitochondrial damage in those models. We thus tested the activity of ABT-263, a dual Bcl-2/Bcl-X inhibitor, to restore I-BET762-mediated apoptosis in all resistant B-cell lymphoma cell lines. The E*μ*-*myc/Bcl-2* and U2932-4RH models showed a high sensitivity to the drug with LD_50_ concentrations, at 48 and 240 h respectively, of approximately 18 nM (data not shown). Exposure of E*μ*-*myc/Bcl-2* and U2932-4RH lymphomas to ABT-263 LD_50_ in combination with the respective I-BET762 LD_70_ concentrations showed significant loss of mitochondrial membrane potential, cell viability and an increase in DNA fragmentation (Figures 4a and b,Supplementary Figure S3b and Supplementary Table S4). In line with these results, protein analysis revealed that combination treatment in E*μ*-*myc/bcl-2* and U2932-4RH lymphomas displayed aberrations in Myc and Bcl-2 expression as well as increased caspase activation (Figure 4c).

As demonstrated in Figure 3g, Raji-4RH and RL-4RH lymphomas do not express Bcl-2 and, for this reason, ABT-263 treatment resulted inefficacious, either as a single agent or in combination, even at high micromolar doses (Supplementary Figure S4).

**Figure 5:**
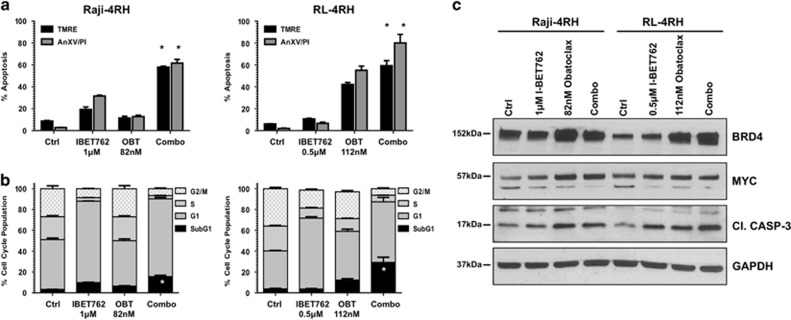
Combination of I-BET762 with obatoclax increases apoptosis in rituximab/chemoresistant B-cell lymphomas. (**a** and **b**) Human Raji-4RH and RL-4RH were treated with indicated concentrations of obatoclax or I-BET762 for 240 h. Apoptosis was assessed by flow cytometric analysis of TMRE, surface exposure of phosphotidylserine (annexin V staining) and cell-cycle analysis. Each point represents the mean ±S.E. value of three individual experiments, *P<0.05. (**c**) Western blot analysis on whole-cell lysates prepared from the human Raji-4RH and Rl-4RH lymphomas treated for 72 h with the single agents or in combination. The expression of indicated proteins was detected with specific antibodies and loading normalization was confirmed by GAPDH probing

With this, we proposed that other alterations in the delicate balance between anti- and proapoptotic proteins may have occurred and be responsible for the resistance to I-BET762 treatment. To examine this, we took advantage of a broad-spectrum BH3-only mimetic compound, obatoclax. We calculated LD_50_ concentrations for the small-molecule inhibitor in Raji-4RH (82 nM) and RL-4RH (112 nM) lymphomas after 10 days of exposure. Combination treatment of Raji-4RH and RL-4RH lymphomas with obatoclax with I-BET762 resulted in marked induction in loss of mitochondrial membrane potential, concurrent with increased exposure of phosphotidylserine and increased DNA fragmentation compared with single treatments (Figures 5a and b and Supplementary Table S4). Western blot analysis following all treatments showed that obatoclax and combination modestly increased BRD4 and Myc protein expression. Further, a moderate increase in activated caspase-3 was observed which is in line with our other apoptotic readouts (Figure 5c). These results suggest that combination of BETI with BH3-only mimetic compound can represent a valid therapeutic strategy for lymphoma patients carrying genetic alterations affecting mitochondrial response to apoptotic stimuli.

## Discussion

Aggressive hematological malignancies including B-cell lymphomas commonly involve deregulation of Myc oncogenic activity. Increased Myc expression is correlated with poorer outcome in patients treated with standard of care therapies including rituximab and chemotherapy. Owing to inferior responses of these patients to standard of care treatments, novel therapeutic approaches are urgently required. BETI are promising therapeutic compounds that efficiently target BET bromodomains, leading to inhibition of critical oncogenic proteins, including Myc.^4, 5, 6, 12^ BETI including OTX-015 and I-BET762 have entered phase I clinical trials in patients with varying solid and hematological malignancies.^12, 23^ Currently, no data are available for I-BET762, but a recent report of data from a phase I trial with OTX-015 indicated clinical responses in leukemia and lymphoma patients in the absence of major adverse toxicities.^12^ This exciting clinical data demonstrate promise for BETI as a potent anticancer therapy, but currently, the antitumor mechanisms induced by BETI are currently not well understood. Dissecting mechanisms required by BETI to mediate strong clinical responses, including apoptosis, would provide strong direction on how to best utilize BETI as monotherapy or in novel combination treatment strategies.

With this aim we took advantage of primary and derived compound mutant murine lymphomas from the E*μ*-*myc* transgenic mouse model of B-cell lymphoma^18, 24, 25^ and human B-cell lymphoma lines with matched isogenic chemotherapy/rituximab resistant cell lines.^26^ To delineate the pathways required by BETI, we used I-BET762 compound, a potent inhibitor of BET bromodomain proteins,^13^ and evaluated its ability to mediate apoptosis in the different models. In line with previous reports demonstrating that treatment of human and mouse cancer models with BETI, including I-BET762, I-BET151 or JQ1, induce apoptosis,^4, 6, 11^ we show that I-BET762 mediates apoptosis of murine and human B-cell lymphoma cell lines *in vitro* involving mitochondrial membrane perturbation, caspase activation and DNA fragmentation. Our data indicate that the mitochrondrial apoptotic pathway is important for BETI-mediated apoptosis. Anticancer agents frequently engage the p53 pathway to activate the mitochondrial apoptotic pathway.^14^ However, epigenetic therapies such as HDAC inhibitors have been shown to induce mitochondrial mediated apoptosis independent of p53 signaling.^18, 25^ Recent reports indicate that dependence of p53 signaling for BETI-mediated apoptosis may be cell context dependent. In models of acute lymphoblastic leukemia and glioblastoma, BETI induced cell death independently of p53 signaling.^15^ However, in acute myeloid leukemia cells harboring mutations in DMNT3A, BETI combination with known activators of p53, induced cell death via a p53 mediated pathway.^16^ Using E*μ*-*myc* lymphomas, deficient for p53 or its activator p19^Arf^, we show that a functional p53 pathway was not necessary for the apoptosis-inducing effects of I-BET762.

Loss of apoptosome function is observed in many cancers, and has been implicated as an underlying mechanism of chemo-resistance.^27, 28^ Specifically, it was shown that cell lines derived from Burkitt lymphomas were found to be resistant to apoptosis induction by anticancer agents due to insufficient levels of Apaf1 in the cytosol.^28^ Moreover, loss of Apaf1 in E*μ*-*myc* lymphomas (E*μ*-*myc*/*apaf1*^−/−^) remained sensitive to dexamethasone and etoposide, though induction of apoptosis was delayed.^29^ Although, E*μ*-*myc/apaf1*^−/−^ lymphomas treated with I-BET762 displayed severe attenuation of DNA fragmentation and complete abrogation of caspase activation, induction of phosphotidylserine exposure as well as loss of mitochondrial membrane potential and clonogenic potential still occurred. Further, E*μ*-*myc/apaf1*^−/−^ lymphomas displayed loss of LCB-I with concurrent gain of LCB-II, a biochemical feature of autophagy.^19^ This intriguing result is similar to previous data showing the ability of HDAC inhibitors to induce autophagy in the same E*μ*-*myc/apaf1*^−/−^ lymphoma model.^18^ This work also demonstrated that HDAC inhibitor induced autophagy mediated significant *in vivo* therapeutic efficacy in mice bearing E*μ*-*myc/apaf1*^−/−^ lymphomas. Whether this ability of BETI and HDAC inhibitors to induce autophagy-mediated cell death in the absence of a functional apoptosome is a feature of all epigenetic-related therapies remains to be determined. However, these exciting results highlight the potential of BETI to induce clinical responses in otherwise non-responsive B-cell lymphomas with aberrations in apoptosome function.

As stated, we identified that I-BET762 engages the mitochondrial apoptotic pathway in murine and human B-cell lymphomas. Blockade of mitochondrial damage through forced overexpression of Bcl-2 abrogated the ability of I-BET762 to kill E*μ*-*myc* lymphomas and inhibit their clonogenic ability, although cell-cycle arrest and growth inhibition were still evident. Interestingly, U2932 tumor cells, despite the high Bcl-2 protein expression, displayed high sensitivity to I-BET762 treatment and the mitochondrial membrane integrity was completely lost after treatment. Indeed, while the U2932 model expresses an endogenous BCL2 protein, in the E*μ*-myc/Bcl-2 model, Bcl-2 gene is ectopically expressed under the control of a constitutively active promoter (CMV) that cannot be regulated by any transcriptional regulator, including BRD4. The E*μ*-myc/Bcl-2 model has in fact been used in this paper as a general model of mitochondrial protection.

A link was previously demonstrated between induction of the mitochondrial apoptotic pathway by HDAC inhibitors and therapeutic efficacy using the E*μ*-*myc* lymphoma model.^18, 25^ Further, combination of HDAC inhibitors with ABT-737, a small-molecule inhibitor of prosurvival Bcl-2 proteins Bcl-2, Bcl-X and Bcl-w, resulted in synergistic mediated apoptosis of E*μ*-*myc* lymphomas overexpressing Bcl-2 or Bcl-X, but not Mcl-1.^30^ We demonstrated that indeed combination of I-BET762 with ABT-263,^31^ a similar inhibitor as ABT-737, overcame Bcl-2 mitochondrial blockade and restored I-BET762-mediated apoptosis in E*μ*-*myc/Bcl-2* lymphomas. This exciting result led us to investigate this combination in all three human B-cell lymphomas that were resistant to I-BET762-induced apoptosis. As expected, I-BET762 combination with ABT-263 resulted in apoptosis only in the U2392-4RH (which overexpress both Myc and Bcl-2) while Raji-4RH and RL-4RH cell lines remained resistant. Instead, combination treatment with I-BET762 and obatoclax,^32^ a small-molecule inhibitor of a wide number of Bcl-2 proapoptotic family members, restored apoptosis in Raji-4RH and RL-4RH B-cell lymphomas. In these models, obatoclax treatment has been described to induce an increased expression of Puma and Noxa levels, thus leading to cell death.^26^ These data further confirm the importance of mitochondrial damage as a key event in I-BET762-mediated apoptosis.

In conclusion, we showed that the clinical relevant BET bromodomain inhibitor I-BET762 induced apoptosis of murine and human B-cell lymphomas via the mitochondrial apoptotic pathway. Inactivation of the p53 and apoptosome-signaling pathway did not abrogate features of apoptosis. Cells deficient in apoptosone function displayed a delay in the therapeutic response associated with biochemical features of autophagy. Finally, combination studies illustrated the complexity by which protection of mitochondria protection can arise in rituximab and chemotherapy-resistant lymphomas. These results are highly significant as they add vital knowledge for clinical decisions regarding precision medicine strategies for using BET inhibitors as a single agent or in combination to treat patients with aggressive treatment refractory B-cell lymphomas.

## Materials and Methods

### Cell culture and reagents

E*μ*-*myc*, E*μ*-*myc*/*p53*^−/−^, E*μ*-*myc*/*p19*^*Arf*−/−^, E*μ*-*myc*/*Apaf1*^−/−^ and E*μ*-*myc*/*Bcl-2* B-cell lymphomas were kindly provided by Ricky W Johnstone. E*μ*-*myc* lymphomas were cultured in six-well plates in high-glucose Dulbecco's modified Eagle's medium supplemented with 10% fetal calf serum, penicillin/streptomycin, 0.1 mM l-asparagine and 50 *μ*M 2-mercaptoethanol in a 37 °C, 10% CO2 humidified incubator. All human B-cell lymphoma cell lines (RAJI, RL, U2932, RAJI-4RH, RL-4RH and U2932-4RH) were kindly provided by Francisco J Hernandez-Ilizaliturri and cultured under standard conditions (37 °C, with 5% CO2) in RPMI (Roswell Park Memorial Institute) 1640 medium supplemented with 10% fetal bovine serum (FBS) and penicillin/streptomycin. I-BET762 was purchased from XcessBio (San Diego, CA, USA) as a 10 mM DMSO solution. ABT-263 was purchased from ChemieTek (Indianapolis, IN, USA) and dissolved in DMSO for the preparation of stock solutions (10 mM). Obatoclax was purchased from SelleckChem (Houston, TX, USA) as a 10 mM DMSO solution.

### *In vitro* cell death and proliferation assays

E*μ*-*myc* lymphoma cells were seeded in 24-well plates to a final concentration of 1.5 × 10^4^ cells/well. The day after, the selected compounds were added at the indicated concentrations. Viability tests were performed by PI uptake (Sigma Aldrich), Annexin V staining (BD Pharmingen, San Jose, CA, USA), cell-cycle analysis or tetramethylrhodamine ethyl ester (TMRE) staining (Sigma Aldrich) according to the manufacturer's instructions. Proliferation assays were performed using the MTS reagent (Promega, Madison, WI, USA) prepared according to the manufacturer's instructions.

### Western blotting and antibodies

Whole-cell lysates were prepared in RIPA buffer (Sigma Aldrich) supplemented with phosphatase and protease inhibitors (Roche, Indianapolis, IN, USA). Protein quantification was performed with the DC Protein Assay kit (Bio-Rad, Hercules, CA, USA) and 20 *μ*g of proteins were loaded in 4–15% Mini-PROTEAN precast gels (Bio-Rad). The following primary antibodies were used for western blotting: anti-MYC (Epitomics, Burlingame, CA, USA; rabbit, 1:2000), anti-BRD4 (Epitomics; rabbit, 1 : 1000), BCL2 (Cell Signaling Technology, Danvers, MA, USA; rabbit, 1 : 2000), GAPDH (Cell Signaling Technology; rabbit, 1 : 5000), Cleaved-Caspase3 (Cell Signaling Technology; rabbit, 1 : 2000), LC3B (Cell Signaling Technology; rabbit, 1 : 1000), Bak (Cell Signaling; 1 : 1000). Each western blot data have been confirmed in at least two biological independent experiments.

### Clonogenic assays

Cells were pretreated for 48 h with 500 nM of I-BET762 in a 24-wells plate, counted and added to 6 ml of soft agar (20% FBS, 0.35% agar in media) at a concentration of 1 × 10^3^ cells/ml. A bottom layer of 0.7% soft agar was plated in six-wells plate and allowed to solidify at room temperature for 30 min. The mixture of agar and cells was then added on top of the bottom layer (2 ml/well) and allowed to set at room temperature for 30 min. Plates were incubated in a humidified 37 °C incubator for 12 days and colonies were counted using an inverted microscope.

### Statistical analysis

Data were displayed as mean±S.E.M. calculated from at least three biological replicates from independent experiments. Differences were determined using two-tailed unpaired *t*-tests and two-way ANOVA, using GraphPad Prism software. *P*-values less than 0.05 were assigned statistically significant.

## Abbreviations

BET: bromodomain and extraterminal domain family
BETI: BET bromodomain inhibitor
BRD4: BET bromodomain 4
PI: propidium iodide
