# Supplementary material for: Mitochondrial protection impairs BET bromodomain inhibitor-mediated cell death and provides rationale for combination therapeutic strategies
Source: Cell Death Dis. 2015 Dec 10;6(12):e2014–. doi: 10.1038/cddis.2015.352 (PMC4720887; doi:10.1038/cddis.2015.352)
Supplement: Supplementary Figures and Tables [file cddis2015352x1.pdf]

Supplement Figure 1

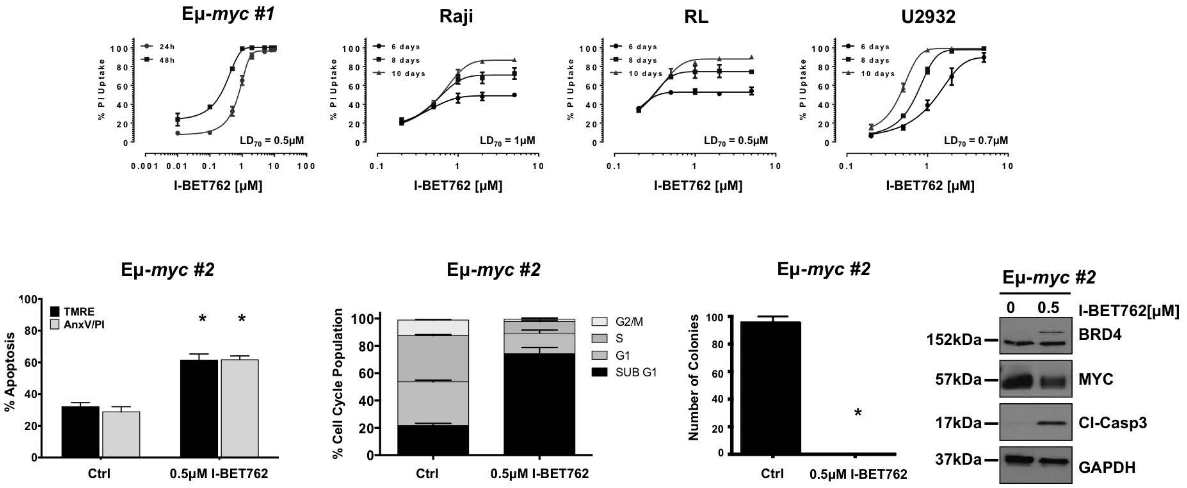

Supplement Figure 2

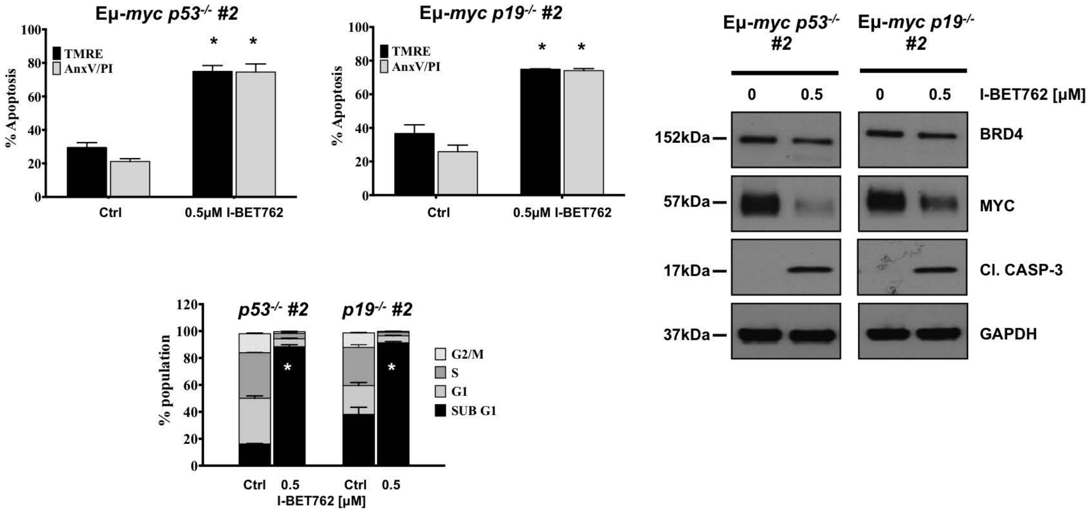

Supplement Figure 3

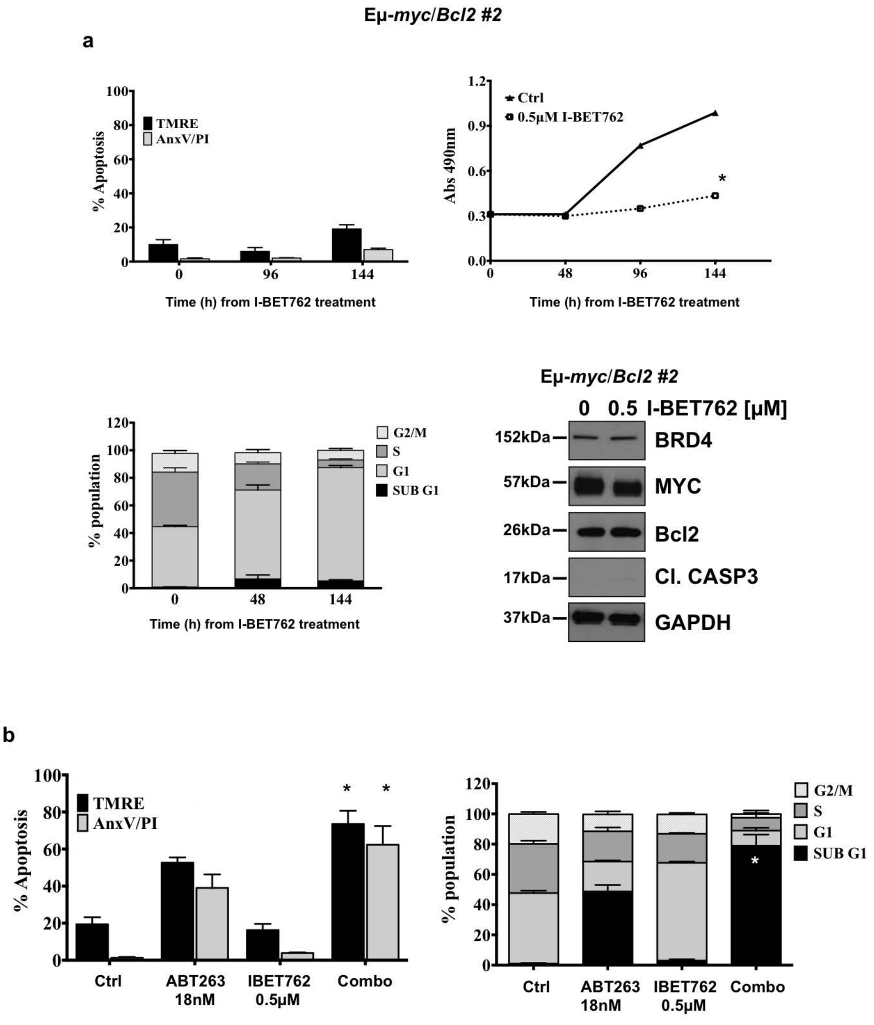

Supplement Figure 4

Raji-4RH

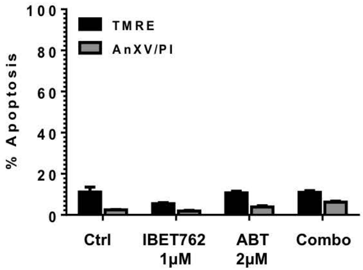

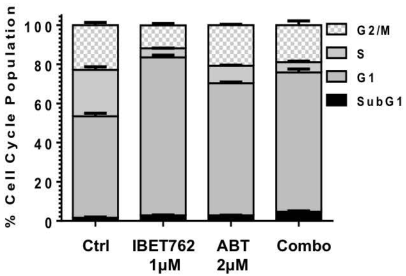

RL-4RH

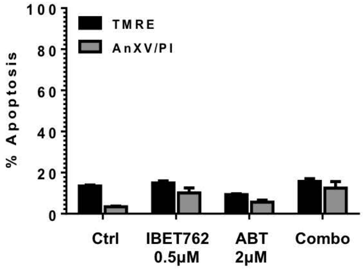

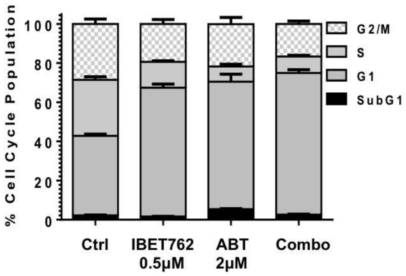

Table 1: Cell cycle analysis summary of Eμ-*myc* B-cell lymphomas following treatment with IBET762 for 48 hours

#1 Eμ-myc lymphoma	SubG1	G1	S	G2M
Ctrl	28.1 (5.1)	30.6 (2.6)	29.6 (2.9)	10.2 (1.2)
0.5μM IBET762	88.2 (2.7)	2.6 (1.6)	4.9 (1.2)	1.1 (0.4)
**#2 Eμ-myc lymphoma**				
Ctrl	21.8 (1.5)	31.9 (1.2)	33.9 (0.7)	11.2 (0.4)
0.5μM IBET762	74.2 (4.5)	15.02 (2.4)	8.6 (1.0)	1.7 (0.9)
**#1 Eμ-myc/p53-/- lymphoma**				
Ctrl	6.2 (2.04)	39.7 (0.9)	35.9 (0.2)	16.6 (1.6)
0.5μM IBET762	78.4 (1.7)	14.1 (0.9)	5.5 (0.4)	1.8 (0.6)
**#2 Eμ-myc/p53-/- lymphoma**				
Ctrl	16.1 (0.3)	34.0 (1.7)	33.8 (0.3)	14.1 (0.5)
0.5μM IBET762	88.3 (1.5)	5.9 (0.5)	3.9 (0.6)	1.3 (0.3)
**#1 Eμ-myc/p19-/- lymphoma**				
Ctrl	20.3 (2.4)	31.1 (0.4)	33.9 (1.7)	13.1 (0.5)
0.5μM IBET762	91.8 (2.5)	3.9 (1.1)	3.5 (1.1)	0.6 (0.2)
**#2 Eμ-myc/p19-/- lymphoma**				
Ctrl	38.0 (5.3)	21.5 (2.3)	28.3 (2.0)	10.8 (0.3)
0.5μM IBET762	91.2 (0.9)	5.3 (0.4)	2.7 (0.3)	0.6 (0.1)

Table 2: Cell cycle analysis summary of human B-cell lymphomas following treatment with IBET762 for 10 days

Raji	SubG1	G1	S	G2M
Ctrl	10.1 (0.3)	62.8 (0.1)	13.7 (1.01)	13.7 (1.3)
1μM IBET762	45.9 (5.2)	41.4 (3.6)	3.6 (0.3)	8.8 (1.4)
**Raji 4RH**				
Ctrl	3.1 (0.2)	45.5 (2.5)	27.9 (0.8)	23.9 (2.1)
1μM IBET762	15.7 (6.5)	73.5 (7.3)	2.5 (0.2)	8.2 (0.7)
**RL**				
Ctrl	7.8 (0.5)	66.6 (1.2)	13.3 (0.8)	12.3 (0.05)
0.5μM IBET762	64.3 (5.2)	30.6 (4.4)	2.5 (0.8)	2.6 (0.6)
**RL 4RH**				
Ctrl	3.6 (0.2)	41.3 (3.4)	29.5 (0.2)	25.6 (3.3)
0.5μM IBET762	4.9 (0.2)	71.7 (1.3)	7.4 (0.3)	15.9 (1.0)
**U2932**				
Ctrl	9.6 (2.1)	59.4 (3.8)	19.1 (0.7)	12.3 (1.2)
0.7μM IBET762	61.7 (2.5)	22.9 (2.8)	7.8 (0.3)	7.5 (0.7)
**U2932 4RH**				
Ctrl	9.9 (1.9)	55.7 (2.1)	17.3 (1.2)	17.4 (1.7)
0.7μM IBET762	28.1 (1.6)	56.5 (1.8)	3.9 (0.1)	11.5 (0.4)

Table 3: Cell cycle analysis summary of Eμ-*myc* B-cell lymphomas

#1 Eμ-myc/apaf1-/- lymphoma	SubG1	G1	S	G2M
0h	1.8 (0.4)	42.2 (1.7)	39.3 (0.9)	14.2 (1.7)
48h	10.9 (3.6)	56.5 (3.9)	23.3 (1.7)	7.4 (1.7)
144h	18.8 (0.5)	57.4 (2.8)	10.3 (1.5)	13.6 (1.6)
**#1 Eμ-myc/Bcl2 lymphoma**				
0h	0.8 (0.1)	43.9 (0.9)	39.6 (3.1)	13.5 (2.0)
48h	6.8 (2.8)	64.4 (3.7)	19.4 (1.3)	8.7 (2.6)
144h	5.3 (0.7)	82.4 (1.3)	5.2 (0.7)	7.2 (1.4)
**#2 Eμ-myc/Bcl2 lymphoma**				
0h	2.6 (0.8)	48.6 (2.2)	35.9 (0.9)	10.7 (1.4)
48h	10.9 (3.1)	73.9 (3.3)	8.5 (1.6)	5.8 (0.7)
144h	3.8 (1.3)	78.5 (0.1)	3.8 (0.03)	13.9 (1.2)

Table 4: Cell cycle analysis summary of combination treatment of Eμ-*myc* lymphomas and human B-cell lymphomas

#1 Eμ-myc/Bcl2 lymphoma	SubG1	G1	S	G2M
Ctrl	1.03 (0.4)	45.9 (1.7)	32.2 (1.9)	21.1 (0.4)
IBET762	3.5 (0.5)	64.2 (1.2)	19.2 (0.5)	13.5 (1.3)
ABT263	52.7 (2.7)	20.4 (0.7	17.6 (0.9)	9.3 (1.03)
Combination	87.5 (1.9)	7.2 (1.1)	3.4 (0.5)	1.8 (0.4)
**#2 Eμ-myc/Bcl2 lymphoma**				
Ctrl	1.9 (0.8)	42.4 (2.4)	27.9 (1.8)	27.7 (2.8)
IBET762	6.9 (2.3)	64.8 (2.5)	12.8 (0.2)	15.7 (1.02)
ABT263	49.6 (4.9)	18.3 (0.3)	17.1 (2.4)	14.9 (2.3)
Combination	86.4 (1.2)	7.3 (0.6)	3.5 (0.5)	2.8 (0.2)
**U2932 4RH**				
Ctrl	3.4 (0.4)	54.5 (1.3)	27.1 (1.4)	14.2 (0.3)
IBET762	11.7 (1.7)	63.9 (0.1)	8.02 (1.01)	15.7 (0.6)
ABT263	19.3 (1.7)	41.7 (2.4)	17.1 (1.9)	21.1 (1.9)
Combination	44.7 (2.1)	26.2 (0.02)	13.3 (1.8)	15.7 (0.3)
**Raji 4RH**				
Ctrl	1.6 (0.3)	51.8 (1.6)	23.7 (1.5)	22.8 (1.4)
IBET762	2.9 (0.1)	80.7 (1.1)	4.6 (0.1)	11.7 (0.9)
ABT263	2.8 (0.1)	67.5 (0.6)	8.9 (0.3)	20.7 (0.4)
Combination	4.6 (0.4)	71.1 (1.8)	5.2 (0.5)	18.9 (2.2)
**Raji 4RH**				
Ctrl	3.2 (0.2)	47.9 (1.4)	21.9 (1.1)	26.9 (2.7)
IBET762	9.8 (0.3)	78.1 (0.2)	3.3 (0.2)	8.7 (0.1)
OBATOCLAX	6.5 (0.4)	43.6 (1.6)	22.9 (1.0)	27.02 (2.9)
Combination	15.6 (1.2)	74.6 (1.4)	3.1 (0.1)	6.6 (0.7)
**RL 4RH**				
Ctrl	2.3 (0.1)	40.6 (0.9)	28.6 (1.6)	28.5 (2.6)
IBET762	1.6 (0.1)	65.8 (1.8)	13.2 (0.5)	19.3 (2.3)
ABT263	5.4 (0.3)	65.1 (3.8)	7.7 (1.2)	21.7 (3.4)
Combination	2.6 (0.2)	72.3 (1.7)	8.4 (0.6)	16.6 (1.5)
**RL 4RH**				
Ctrl	3.8 (0.5)	36.4 (0.5)	23.9 (0.7)	35.9 (1.2)
IBET762	3.6 (0.5)	67.9 (1.4)	9.7 (0.4)	17.2 (0.9)
OBATOCLAX	12.2 (1.3)	46.8 (2.0)	12.2 (0.2)	25.7 (1.2)
Combination	29.3 (4.9)	58.1 (4.2)	6.3 (0.1)	6.4 (0.8)
