# Supplementary material for: Mitochondrial protection impairs BET bromodomain inhibitor-mediated cell death and provides rationale for combination therapeutic strategies
Source: Cell Death Dis. 2015 Dec 10;6(12):e2014–. doi: 10.1038/cddis.2015.352 (PMC4720887; doi:10.1038/cddis.2015.352)
Supplement: Supplementary Information [file cddis2015352x2.doc]

**Mitochondrial protection inhibits BET bromodomain mediated cell death and provides rationale for combination therapeutic strategies**

Elena Lasorsa^1^, Matthew Smonksey^1^, Jason S. Kirk^1^, Zafardjan Dalimov^1^, Spencer Rosario^1,3^, Francisco J. Hernandez-Ilizaliturri^2^, Leigh Ellis^1,3,#^

^1^Department of Pharmacology and Therapeutics, Roswell Park Cancer Institute, Buffalo NY

^2^Department of Medicine, Roswell Park Cancer Institute, Buffalo NY

^3^Department of Molecular Pharmacology and Cancer Therapeutics, State University of New York at Buffalo, NY

**Supplement Figures and Figure Legends**

**Supplement Figure 1: (Upper panels)** Eμ-*myc*, RAJI, RL and U2932 B-cell lymphoma were treated with increasing concentrations of I-BET762 over time. Cell membrane permability was assessed by staining with propidium iodide and measured by flow cytometry. The lethal dose which kills 70% of lymphomas (LD_70_) doses were calculated at 48 hours for the Eμ-*myc* model and at 240 hours for the human lymphoma models (Eμ-*myc* 0.5µM, RAJI 1µM, RL 0.5µM and U2932 0.7µM). **(Lower panels)** A second independent Eμ-*myc* lymphoma was treated with 0.5µM of I-BET762 for 48 hours. Apoptosis was assessed by flow cytometric analysis of TMRE, surface exposure of phosphotidylserine (annexin V staining), and cell cycle analysis. Each point represents the mean value ±SE of individual experiments, *p<0.05. Clonogenic assay on Eμ-*myc*lymphomas after being pre-treated for 48 hours with DMSO or I-BET762 and then seeded in soft agar. Colonies have been counted after 12 days in culture. Each point represents the mean ±SE value of individual experiments, *p<0.05. Western Blot analysis on whole cell lysates prepared from mouse and human lymphoma cell lines treated respectively for 24 and 72 hours with corresponding LD_70_ concentrations of I-BET762 or DMSO. The protein expression of BRD4, Myc, and cleaved caspase 3 were assessed. GAPDH was used as loading control.

**Supplement Figure 2:** Second independent Eμ-*myc/p53*^-/-^ #2 and Eμ-*myc/p19^Arf^*^-/-^ #2lymphomas were treated for 48 hours with 0.5µM I-BET762. (A) Apoptosis was assessed by flow cytometric analysis of TMRE, surface exposure of phosphotidylserine (annexin V staining), and cell cycle analysis. Each point represents the mean ±SE value of individual experiments, *p<0.05. Western blot analysis on whole cell lysates prepared from Eμ-*myc/p53*^-/-^ #2 and Eμ-*myc/p19^Arf^*^-^ #2 lymphomas treated for 24 hours with 0.5µM of I-BET762 or DMSO. Expression of BRD4, Myc, and cleaved caspase3 was assessed. GAPDH was used as loading control.

**Supplement Figure 3: (a)** A second independent Eμ-*myc/bcl-2* #2 lymphoma was treated as indicated with 0.5µM I-BET762. Apoptosis was assessed by flow cytometric analysis of TMRE, surface exposure of phosphotidylserine (annexin V staining), and cell cycle analysis. (E) MTS assay to evaluate the proliferation rate of all four lymphoma models in presence or absence of the I-BET762 over time. Each point represents the mean value of individual experiments ±SE. Western Blot analysis on whole cell lysates prepared from Eμ-*myc/Bcl2* #2 lymphoma treated for 24 hours. Expression of BRD4, Myc, Bcl-2 and cleaved caspase3 was assessed. GAPDH was used as loading control. **(b)** Eμ-*myc/Bcl2* #2 lymphoma was treated with indicated concentrations of ABT-263 or I-BET762 for 48 hours. Apoptosis was assessed by flow cytometric analysis of TMRE, surface exposure of phosphotidylserine (annexin V staining), and cell cycle analysis. Each point represents the mean ±SE value of individual experiments, *p<0.05.

**Supplement Figure 4:** Human Raji-4RH and RlL-4RH lymphomas were treated with respective LD_70_ concentrations of I-BET762 in combination with 2µM of ABT-263 for 240 hours. Apoptosis was assessed by flow cytometric analysis of TMRE, surface exposure of phosphotidylserine (annexin V staining), and cell cycle analysis. Each point represents the mean ±SE value of individual experiments.
